# Lauren classification combined with HER2 status is a better prognostic factor in Chinese gastric cancer patients

**DOI:** 10.1186/1471-2407-14-823

**Published:** 2014-11-07

**Authors:** Miaozhen Qiu, Yixin Zhou, Xinke Zhang, Zixian Wang, Fang Wang, Jianyong Shao, Jiabin Lu, Ying Jin, Xiaoli Wei, Dongsheng Zhang, Fenghua Wang, Yuhong Li, Dajun Yang, Ruihua Xu

**Affiliations:** Department of Medical Oncology, Sun Yat-Sen University Cancer Center; State Key Laboratory of Oncology in South China; Collaborative Innovation Center for Cancer Medicine, 651 Dongfeng Road East, Guangzhou, 510060 China; The Sidney Kimmel Comprehensive Cancer Center, The Johns Hopkins University School of Medicine, Baltimore, MD 21231 USA; Department of pathology, Sun Yat-Sen University Cancer Center; State Key Laboratory of Oncology in South China; Collaborative Innovation Center for Cancer Medicine, 651 Dongfeng Road East, Guangzhou, 510060 China; Faculty of medical sciences, Sun Yat-sen University, Guangzhou Zhongshaner Road No. 74, Guangzhou, China; Department of Molecular Pathology, Sun Yat-Sen University Cancer Center; State Key Laboratory of Oncology in South China; Collaborative Innovation Center for Cancer Medicine, 651 Dongfeng Road East, Guangzhou, China; Department of Experimental Research, Sun Yat-Sen University Cancer Center; State Key Laboratory of Oncology in South China; Collaborative Innovation Center for Cancer Medicine, 651 Dongfeng Road East, Guangzhou, 510060 China

**Keywords:** Gastric cancer, Lauren classification, Human epidermal growth factor receptor 2, Prognosis

## Abstract

**Background:**

Lauren-classification and human epidermal growth factor receptor 2 (HER2) status are two important pathological features of gastric cancer patients. The prognostic value of HER2 in gastric cancer remains controversial. Intestinal type gastric cancer has better prognosis and higher HER2 positive proportion. What is the interaction between these two factors? We hypothesized that a combination of Lauren-classification and human epidermal growth factor receptor 2 (HER2) status (L-H status) might be more meaningful than either factor alone.

**Methods:**

We collected 838 gastric cancer patients at all stages who had received treatment in our cancer center. This study was registered in the website of ClinicalTrials.Gov, with the number NCT01927146. We divided the patients into six groups according to their L-H status: Group A, HER2 negative and intestinal type; Group B, HER2 positive and intestinal type; Group C, HER2 negative and diffuse type; Group D, HER2 positive and diffuse type; Group E, HER2 negative and mixed type; and Group F, HER2 positive and mixed type.

**Results:**

Diffuse type and intestinal type accounted for 51.0% and 33.9%, respectively. The proportion of HER2 positive patients was 11.2%, 25.4%, 2.1% and 10.2% in the whole patient group, intestinal, diffuse and mixed type, respectively. Median overall survival was 34.0 months, 25.3 months, 27.6 months, 19.2 months, 25.9 months and 26.4 months in the six groups patients, P = 0.053. There was a significant difference in survival among the first four groups (P < 0.001). HER2 was an independent prognostic factor in the intestinal type and in stage I + II patients, but not in the diffuse type or stage III + IV patients. L-H status was an independent prognostic factor in patients at all stages. For the diffuse and intestinal types, the multivariate analysis showed that HER2 was not an independent prognostic factor, while Lauren classification and L-H status were. Moreover, L-H status was a better prognostic factor than the Lauren classification.

**Conclusions:**

L-H status is a prognostic factor in diffuse and intestinal type patients, but not in the mixed type. Patients with HER2 negative and intestinal type had the best survival, while patients with HER2 positive status and diffuse type had the worst survival.

## Background

Gastric cancer is the second most common cause of cancer-related death worldwide [1]. The incidence of gastric carcinoma varies significantly from one part of the world to another and it is particularly common in Eastern Asia, especially in China [2]. Amplification, overexpression or both, of human epidermal growth factor receptor-2 (HER2, also known as ERBB2), a transmembrane receptor tyrosine kinase, is present in around 6.1–23.0% of gastric cancers [3–5]. In breast cancer, amplification and overexpression of the HER2 gene are associated with poor outcomes, higher mortality, higher recurrence and metastasis [6–8]. However, the prognostic value of HER2 status in gastric cancer remains controversial. Some studies showed that HER2-positive patients had a favorable survival [9–11], while other studies revealed no relationship between HER2 status and survival [4, 12–14]. The majority of the publications showed that a HER2-postive status, measured by immunohistochemistry (IHC) or fluorescence in situ hybridization (FISH), was associated with poor survival and/or clinicopathological characteristics, such as serosal invasion, lymph node metastases, disease stage, or distant metastases [11, 15, 16].

Although the Lauren classification system dates back to 1965, it is still widely accepted and employed by pathologists and physicians today. According to the Lauren classification, gastric adenocarcinomas can be divided into diffuse, intestinal and mixed type [17]. Cohesive cells that form gland-like structures characterize the intestinal type. For the diffuse type, tumor cells lack cell-to-cell interactions and infiltrate the stroma as single cells or small subgroups, leading to a population of non-cohesive, scattered tumor cells [17]. The intestinal-type is more frequent in males and in elderly patients, while the diffuse-type occurs more frequently in women and young patients [18]. Intestinal type patients have better outcomes than patients with diffuse-type tumors [8, 19–21]. However, HER2 positivity is more common in intestinal-type gastric cancer [15]. The higher rate of HER2 positivity and better survival in the intestinal type is controversial. We hypothesized that the combination of the Lauren classification and HER2 status (L-H status) might be more helpful than either factor alone. In this study, we explored the relationship between Lauren classification and HER2 status; moreover, we also analyzed the prognostic value of L-H status.

## Methods

### Patient collection

From January 1996 to December 2006, we collected clinical information retrospectively from gastric cancer patients who received treatment in our cancer center. Patients included in the study met the following criteria: (1) histologically confirmed gastric adenocarcinoma patients that underwent gastrectomy; (2) adequate paraffin-embedded tumor tissue sample for pathological and HER2 status analysis; and (3) complete medical records with regular survival follow-up data. Overall survival (OS) data was present. The exclusion criteria were: (1) age <18 years old; and (2) other malignancy within the last 5 years, except carcinoma in situ of the cervix, or basal cell carcinoma.

All patients were categorized according to the 7^th^ American Joint Committee on Cancer (AJCC) Tumor-Node-Metastasis (TNM) stage.

### Lauren classification

Assignment of histological type was based on the Lauren criteria. The intestinal type was described as a tumor with glandular architecture, resembling colonic carcinoma. The diffuse type was described as a tumor composed of solitary or small clusters of cells, and lacking glandular structures. The mixed type was described as the combination of these two features. Two pathologists reviewed the original diagnostic slides to make a diagnosis of Lauren classification.

### HER2 evaluation

#### Immunohistochemistry (IHC)

For all patients, HER2 expression was detected by IHC. IHC staining was carried out using an anti-HER-2/NEU (4B5) antibody (Ventana Medical Systems, Inc. Tucson, AZ, USA) as the primary antibody against HER2 on a Ventana Benchmark XT automatic staining system, according to the manufacturer’s instructions. The amended HER2 IHC scoring system for gastric cancer proposed by Hoffmann et al. was used as the criteria for scoring the stained slides [22].

#### Fluorescence in situ hybridization (FISH)

*HER2* amplification levels were measured when the result of IHC was 2+. The PathVysion®HER2 DNA Probe kit (LSI®HER2/neu Spectrum Orange™/chromosome 7 centromere probe (CEP) ®17 Spectrum Green) was used to perform FISH analysis, according to the manufacturer’s protocol. A positive result from FISH was defined as a HER2:CEP17 ratio ≥2.

Any case with IHC 3+ or IHC2+/FISH + was considered to be HER2-positive, while cases with IHC 0 or IHC 1+ or IHC 2+/FISH − were considered as HER2-negative, according to criteria of the European Medicines Agency.

#### L-H status

We divided the patients into six groups according to their Lauren classification and HER2 status (L-H Status): Group A, HER2 negative and intestinal type; Group B, HER2 positive and intestinal type; Group C, HER2 negative and diffuse type; Group D, HER2 positive and diffuse type; Group E, HER2 negative and mixed type; and Group F, HER2 positive and mixed type.

#### Statistical analysis

The Statistical Package of Social Sciences 13.0 software performed all the statistical analyses. A P value <0.05 was considered statistically significant. The Kaplan-Meier method was used to estimate OS. For patients who remained alive, data were censored at the date of the last contact. Kaplan-Meier analysis with log-rank testing was used for univariate analysis. OS was defined as the duration between the date of diagnosis and the date of last contact. Variables showing a trend for association with survival (P <0.05) and variables that were known to have prognostic value were selected for submission to a final multivariate Cox proportional hazards model, while variables that were highly associated with others were excluded from the final multivariate model. The chi-square test was used to compare the clinicopathological data.

We compared the -2log likelihood (which was the parameter in the Cox regression) of two different models of multivariate analysis: the smaller the value, the better the model [23].

#### Ethics statement

All patients signed written informed consent for their information to be used for the study. The independent ethics committees at the Cancer Center of Sun Yat-Sen University approved the study. The study was undertaken in accordance with the ethical standards of the World Medical Association Declaration of Helsinki.

This study was registered in the website of ClinicalTrials.Gov with a number of NCT01927146.

## Results

### Patient demographics

The median age of the 838 patients was 59 years (rang: 18 to 86 years); 554 were male and 284 were female. There were 88 stage IV patients at the time of diagnosis who all received gastrectomy to relieve the symptom of obstruction or bleeding. During follow-up, 91 patients developed distant metastasis and 12 patients had local recurrence. Until January 1, 2014, 77 patients had died from gastric cancer.

### Lauren classification

There were 51.0% (427/838) of patients with diffuse type and 33.9% (284/838) patients with the intestinal type. The remaining 127 (15.1%) patients belonged to the mixed type.

The relationship between clinicopathological features and Lauren classification is showed in Table 1. Among the patients who were younger than 60 years old, 269 (62.7%) had the diffuse type, while for patients who were older than 59 years old; only 158 (38.6%) patients had the diffuse type. The ratio of males to females was significantly higher in the intestinal-type than that in the diffuse-type (3.2 *vs*. 1.3; P < 0.001). Patients in stages III and IV had a higher percentage of diffuse type than those in the stages I and II.

**Table 1:** **Baseline characteristics**

	Lauren classification			***P*** # value	HER2 status		***P*** * value
	Diffuse(%)	Intestinal(%)	Mixed(%)		Negative(%)	Positive(%)	
Sex				<0.0			
Male	242 (43.7)	216 (39.0)	96 (17.3)	01	486 (87.7)	68 (12.3)	0.176
Female	185 (64.7)	68 (23.9)	31 (10.9)		258 (90.8)	26 (9.2)	
Age				<0.001			<0.001
≤59	269 (62.7)	104 (24.2)	56 (13.1)		398 (92.8)	31 (7.2)	
>59	158 (38.6)	180 (44.0)	71 (17.4)		346 (84.6)	63 (15.4)	
Stage				<0.001			0.406
I	68 (47.9)	60 (42.3)	14 (9.8)		131(92.3)	11 (7.7)	
II	96 (41.7)	102 (44.3)	32 (13.9)		203 (88.3)	27 (11.7)	
III	215 (56.9)	95 (25.1)	68 (18.0)		335 (88.6)	43 (11.4)	
IV	48 (54.5)	27 (30.7)	13 (14.8)		75 (85.2)	13 (14.8)	
Degree of differentiation				<0.001			<0.001
Well + Moderate	0 (0)	262 (76.2)	82 (23.8)		270 (78.5)	74 (21.5)	
Poor + signet ring cell	427 (86.4)	22 (4.5)	45 (9.1)		474 (96.0)	20 (4.0)	
Location				<0.001			<0.001
Proximal	110 (35.9)	146 (47.7)	50 (16.3)		251 (82.0)	55 (18.0)	
Distal	276 (59.1)	128 (27.4)	63 (13.5)		436 (93.4)	31 (6.6)	
Total stomach	41 (63.1)	10 (15.4)	14 (21.5)		57 (87.7)	8 (12.3)	
Adjuvant chemotherapy							
Yes	302 (51.4)	192 (32.7)	94 (15.9)		531 (90.3)	57 (9.7)	
No	77 (47.5)	65 (40.1)	20 (12.4)	0.170	138 (85.2)	24 (14.8)	0.063

HER2: human epidermal growth factor receptor 2.

#*P* values of Lauren classification in different clinical features. *P values of HER 2 status in different clinical features.

### HER2 status

The percentages of IHC negative, 1+, 2+ and 3+ were 51.2% (429/838), 25.5% (214/838), 15.4% (129/838) and 7.9% (66/838), respectively. For the IHC 2+ patients, 28 patients were diagnosed as FISH positive. Thus, the proportion of patients positive for HER2 was 11.2% (94/838) in the whole group of patients.

Among patients who were older than 60 years, there were more HER2 positive patents than among those younger than 59 years old. Stage IV patients had the highest proportion of HER2 positive (14.8%). The relationship between clinicopathological features and HER2 status is shown in Table 1.

### L-H status

**Figure 1:**
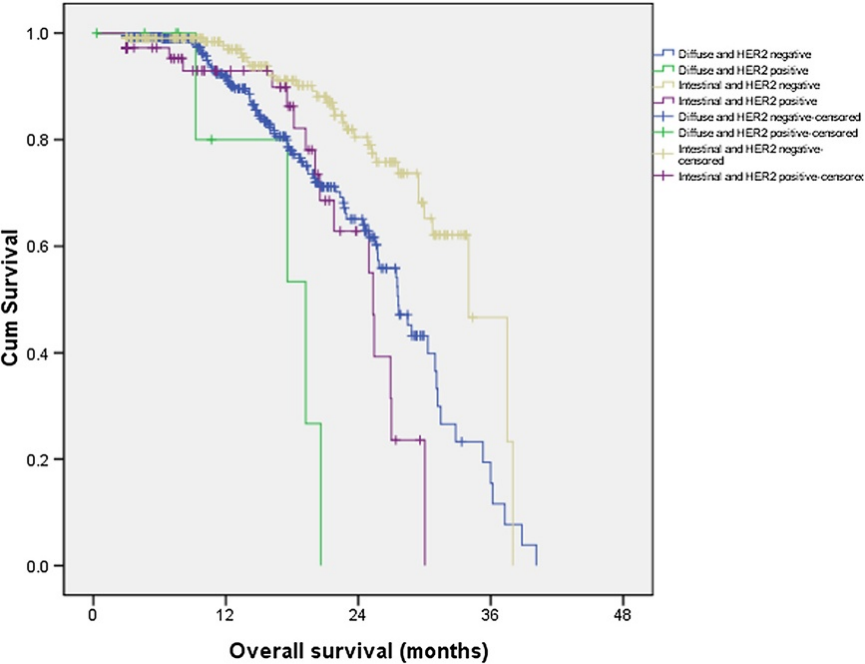
**The survival difference among different L-H status.**

The proportions of HER2 positive patients were 25.4%, 2.1% and 10.2% in the intestinal type, diffuse type and mixed type, respectively (P < 0.001). The median OS (from the time of diagnosis to the time of last contact) was 34.0 months, 25.3 months, 27.6 months, 19.2 months, 25.9 months and 26.4 months in the six groups of patients (P = 0.053). Considering that the mixed type contained the features of diffuse type and intestinal type, the difference between diffuse and intestinal type could not be fully evaluated in the mixed type. In subsequent analyses we only evaluated the value of L-H classification in the diffuse and intestinal types. The number of patients in these four groups was 212, 72, 418 and nine respectively. The median survival was 34.0 months, 25.3 months, 27.6 months and 19.2 months (P < 0.001; Figure 1). For the stage IV patients (including 88 concurrent metastasis and 91 metachronous metastasis patients), if we calculated the survival from the time of metastasis to the time of last contact, the median overall survival was 13.7 months, 10.2 months, 10.8 months and 7.9 months (P = 0.001).

The relationship between L-H status and clinicopathological features is shown in Table 2. From the table, we could conclude that L-H status was a useful index. Among the four L-H groups, the clinicopathological features were quite different, except for the percentage of adjuvant chemotherapy.

**Table 2:** **The relationship between different L-H status and clinical features**

	Group A	Group B	Group C	Group D	***P*** value
Sex					
Male	163	54	235	6	
Female	49	18	183	3	<0.001
Age					
≤59	82	21	264	6	
>59	130	51	154	3	<0.001
Stage					
I	48	10	69	1	
II	80	23	93	2	
III	66	30	211	3	
IV	18	9	45	3	<0.001
Degree of differentiation					
Well + Moderate	193	69	0	0	
Poor + signet ring cell	19	3	418	9	<0.001
Location					
Proximal	99	45	111	1	
Distal	104	24	269	7	
Total stomach	9	3	38	1	<0.001
Adjuvant chemotherapy					
Yes	144	48	297	5	
No	50	15	76	1	0.505

Group A, HER2 negative and intestinal type; Group B, HER2 positive and intestinal type; Group C, HER2 negative and diffuse type; Group D, HER2 positive and diffuse type.

### Survival analysis

**Figure 2:**
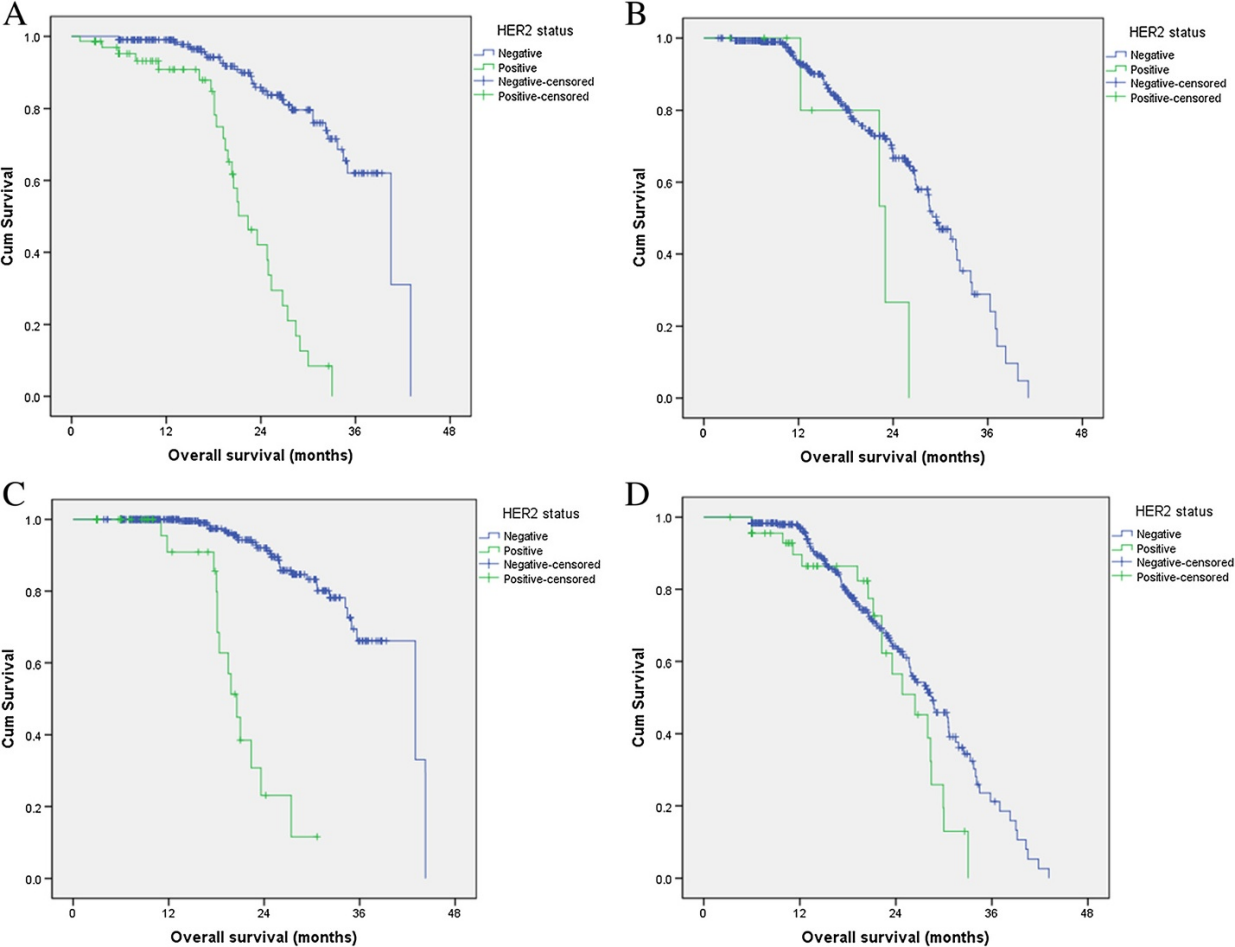
**Kaplan–Meier curves of HER2-positive and -negative patients for overall survival in (A), intestinal type (B), diffuse type (C), TNM stage I/II and (D) TNM stage III/IV.**

Both univariate and multivariable analyses were used to evaluate factors associated with OS. Factors of TNM stage (P < 0.001), degree of differentiation (P = 0.015), Lauren classification (P = 0.006), HER2 status (P = 0.033) and L-H status (P = 0.003) were all significantly associated with OS in the univariate analysis.To further explore the prognostic value of HER2, we analyzed the survival difference between HER2 positive and HER2 negative patients in intestinal type and diffuse type, respectively. We found that HER2 positivity was an independent adverse prognostic factor in the intestinal type (P < 0.001), but not in the diffuse type (P = 0.084; Figure 2A, B).We then analyzed the prognostic value of HER2 positivity in different stages. HER2 positivity was an independent adverse prognostic factor in stage I and II patients (P < 0.001), but not in stage III and IV patients (P = 0.125; Figure 2C, D).

**Figure 3:**
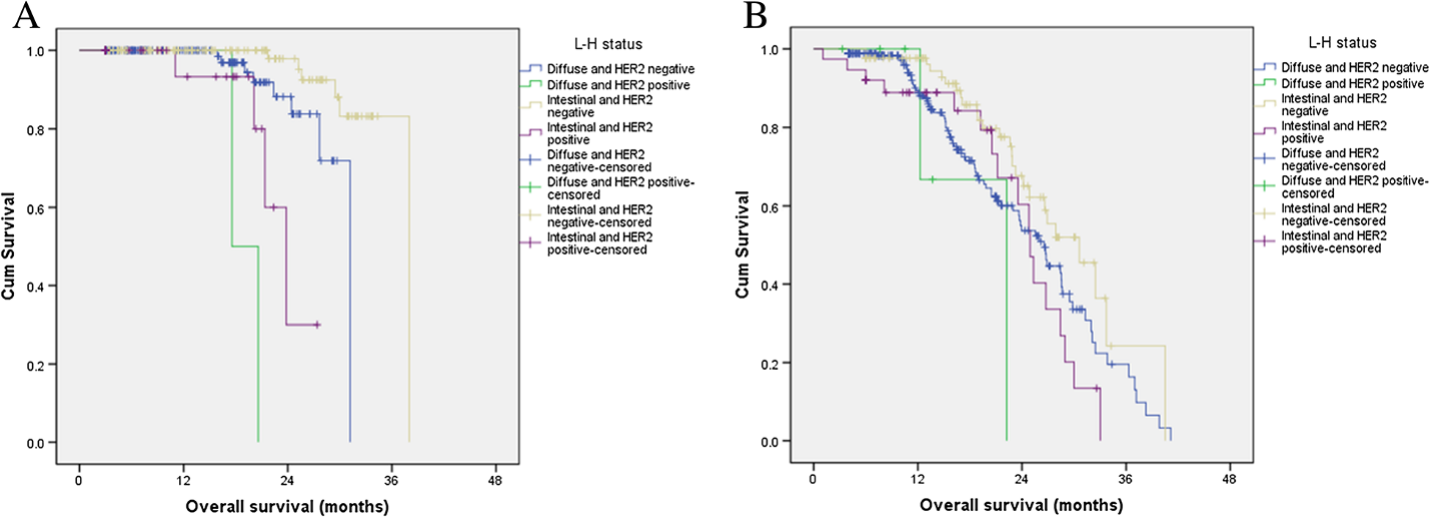
**Kaplan–Meier curves of L-H status for overall survival in (A), TNM stage I/II and (B) TNM stage III/IV.**

For the multivariable regression analysis, we first set up a model (model A) that included age, gender, degree of differentiation, TNM stage, Lauren classification and HER2 status. Model A showed that age, degree of differentiation, TNM stage and Lauren classification were independent factors for OS (*P* = 0.001, 0.017, <0.001 and 0.047, respectively, Table 3). HER2 status was not an independent prognostic factor (P = 0.285). The -2log likelihood was 1663.155. We then set up another model (model B), which was identical to the first one except that the Lauren classification and HER2 status were replaced by the L-H status. In model B, TNM stage and L-H status were independent factors for OS (P = 0.028, <0.001 and 0.006, respectively, Table 3). The -2log likelihood was 1411.610.We also analyzed the prognostic value of L-H status in different stages. L-H status was an independent prognostic factor in both early stage (I and II) patients (P < 0.001) and advanced stage (III and IV) patients (P = 0.036; Figure 3A, B).

**Table 3:** **The multivariable analysis of overall survival in gastric carcinoma**

	Model A			Model B		
	Hazard ratio	95% CI	***P*** value	Hazard ratio	95% CI	***P*** value
Gender	1.208	0.736-1.983	0.455	0.984	0.566-1.711	0.954
Age	2.302	1.383-3.831	0.001	1.472	1.059-2.047	0.028
Stage	3.604	2.551-5.091	<0.001	3.610	2.490-5.233	<0.001
Degree of differentiation	0.505	0.288-0.886	0.017	0.424	0.167-1.074	0.070
Lauren classfication	1.440	1.004-2.066	0.047	–	–	–
HER2 status	0.669	0.320-1.398	0.285	–	–	–
L-H status	–	–	–	2.222	1.259-3.920	0.006

*Abbreviations*: *CI* confidence interval, *HER2* human epidermal growth factor receptor-2, *L-H status* Lauren classification and HER2 status.

Model A includes the factors of Lauren classification and HER2 status; Model B includes the combination factor of L-H status.

## Discussion

The prognostic value of HER2 status in gastric cancer remains controversial. Some studies reported that HER2 positivity was an adverse prognostic factor, while some found that it indicated better survival. Other studies even considered that it had no relationship with survival. Based on the Lauren classification, gastric cancers could be divided into the diffuse type, intestinal type and mixed type. The intestinal type has a better survival than the diffuse type. However, intestinal type patients were more likely to be HER2 than diffuse type patients. In our study, HER2 was not an independent prognostic factor for gastric cancer patients in the multivariate analysis. When we separated the patients into diffuse and intestinal types, we found that HER2 was an independent adverse prognostic factor for the intestinal type. We also analyzed the prognostic value of HER2 positivity in patients at different stages. HER2 positivity was an independent prognostic factor for stage I and II patients, but not in stage III and IV patients. This was different from the result of Kataoka et al. [14]. They analyzed 213 Japanese gastric cancer patients retrospectively and found that the OS of HER2-negative and -positive patients was not significantly different in the whole group patients. However, in patients with stage III/IV, they found that the OS was worse in HER2-positive patients (P = 0.0149) [14]. In the 2012 European Society for Medical Oncology (ESMO) conference, a multicenter study conducted by Kurokawa et al. showed that HER2 positivity was an independent prognostic factor in stage I and II patients, but not in stage III and IV patients [24]. This was consistent with our results. These were all retrospective analyses. Therefore, prospective studies are required to explore the prognostic value of HER2 in early stage gastric cancer patients.

Based on the analysis above, we hypothesized that when we discussed the prognostic value of HER2 positive, there were other factors that should be into consideration, such as the TNM stage and the Lauren classification.

HER2 positivity was much more common in the proximal, intestinal type and stage IV gastric cancer patients. Male, older patients and proximal gastric cancer patients had a higher percentage of intestinal type. These basic clinicopathological features were all consistent with previous studies [4, 15, 16, 18–21].

In the 2014, at the American Society of Clinical Oncology (ASCO) annual meeting, both HER2 positivity and the Lauren classification were considered as the most important progresses in gastric cancer in the last 50 years. These were two important pathological features of gastric cancer. In the present study, we combined these two factors together and proposed the concept of L-H status. Since mixed type was not a pure group, L-H status is not a good option for mixed type. We only considered intestinal and diffuse type in the analysis. We divided the gastric cancer patients according to their L-H status to create four groups: Group A, HER2 negative and intestinal type; Group B, HER2 positive and intestinal type; Group C, HER2 negative and diffuse type; and Group D, HER2 positive and diffuse type. Group C had the largest number of patients. Unsurprisingly, the patients in Group A had the best prognosis, while those in Group D had the worst. Although both intestinal type and L-H status were independent prognostic factors in the multivariate analysis, the -2log likelihood was smaller in the L-H status model: the smaller the value of this statistic, the better the model. Therefore, the L-H status was better than the Lauren classification for predicting the prognosis.

In the multivariate analysis, age, TNM stage and L-H status were all independent prognostic factors for gastric adenocarcinoma patients. The L-H status could replenish the TNM stage. Moreover, we found that L-H status was an independent prognostic factor in stage I + II and stage III + IV patients. Although the L-H status was not useful in the mixed type, we recommend that all the gastric cancer patients should be subjected to Lauren classification and their HER2 status checked to determine their L-H status. It is not only helpful to evaluate prognosis, but also is helpful to decide treatment. For HER2 positive metastasis gastric cancer patients, trastuzumab is the standard treatment.

The limitations of the present study are: 1) its retrospective nature from a single-institution; 2) the fact that the impact of various treatment-related outcomes could not be fully evaluated; and 3) that progression free survival or disease free survival could not be fully analyzed.

External validation using other large databases or prospective studies to evaluate the prognostic effect of L-H status is required. The underlying mechanism of intestinal type gastric cancer and relationship with high HER2 expression requires further exploration.

## Conclusions

In this large sample size study, we found that HER2 positivity was not an independent prognostic factor in the whole group of patients, but it was in the intestinal type and stage I and II patients. The combination of the Lauren classification and HER2 status (L-H status) was a better prognostic factor than the Lauren classification alone in the diffuse and intestinal type. We recommend that all the gastric cancer patients should be subjected to Lauren classification and their HER2 status checked to determine their L-H status.

## Abbreviations

HER2: Human epidermal growth factor receptor-2
IHC: Immunohistochemistry
FISH: Fluorescence in situ hybridization
L-H status: Lauren classification and HER2 status
OS: Overall survival
AJCC: American Joint Committee on Cancer
TNM: Tumor-Node-Metastasis
CEP: Centromere probe
ESMO: European Society for Medical Oncology
ASCO: American Society of Clinical Oncology.
